# Vascular ring anomaly in a patient with phosphomannomutase 2 deficiency: A case report and review of the literature

**DOI:** 10.1002/jmd2.12160

**Published:** 2020-08-19

**Authors:** Zhen Qian, Jef Van den Eynde, Stephane Heymans, Luc Mertens, Eva Morava

**Affiliations:** ^1^ Department of Clinical Genomics Mayo Clinic Rochester Minnesota USA; ^2^ Research Group Experimental Oto‐Rhino‐Laryngology KU Leuven Leuven Belgium; ^3^ Faculty of Medicine KU Leuven Leuven Belgium; ^4^ Labatt Family Heart Center, Department of Paediatrics Hospital for Sick Children and University of Toronto Toronto Ontario Canada; ^5^ Department of Cardiovascular Sciences KU Leuven Leuven Belgium; ^6^ Cardiovascular Research Institute Maastricht (CARIM) Maastricht University Maastricht The Netherlands; ^7^ Netherlands Heart Institute (ICIN) Utrecht The Netherlands

**Keywords:** cardiovascular anomaly, congenital disorder of glycosylation, congenital heart defect, phosphomannomutase 2, vascular ring

## Abstract

**Background:**

Congenital disorders of glycosylation (CDG) are a group of metabolic disorders well known to be associated with developmental delay and central nervous system anomalies. The most common CDG is caused by pathogenic variants in the phosphomannomutase 2 gene (*PMM2*), which impairs one of the first steps of N‐glycosylation and affects multiple organ systems. Cardiac involvement can include pericardial effusion, cardiomyopathy, and arrhythmia, while an association with cardiovascular congenital anomalies is not well studied.

**Case summary:**

We report a 6‐year‐old individual who initially presented with inverted nipples, developmental delay, and failure to thrive at 3 months of age. At 4 months, due to feeding problems, swallowing exam and echocardiography were performed which revealed a vascular ring anomaly based on a right aortic arch and aberrant left subclavian artery. Subsequent whole exome gene sequencing revealed two pathogenic PMM2‐CDG variants (E139K/R141H) and no known pathogenic mutations related to congenital heart defect (CHD).

**Discussion:**

This is the first report of vascular ring anomaly in a patient with PMM2‐CDG. We conducted a literature review of PMM2‐CDG patients with reported CHD. Of the 14 patients with PMM2‐CDG and cardiac malformation, the most common CHD's were tetralogy of Fallot, patent ductus arteriosus, and truncus arteriosus. The potential important link between CDG and CHD is stressed and discussed. Furthermore, the importance of multidisciplinary care for CDG patients including early referral to pediatric cardiologists is highlighted.

## 1 INTRODUCTION

Congenital heart defects (CHDs) affect around 0.9% of newborns and are often found in association with other congenital disorders.^1^ Isolated and syndromal heart defects could be both inherited in a Mendelian fashion, but for many CHD the underlying cause remains unknown and an interplay with several environmental factors is proposed.^2^

Congenital disorders of glycosylation (CDGs) are a large group of metabolic disorders.^3^ Given the importance of glycosylation in the function of several organs such as the immune system, the central nervous system, and the processes involved during organogenesis, CDG has various presentations and affects a wide range of body systems. The most common CDG is phosphomannomutase 2 deficiency (PMM2‐CDG).^4^ PMM2‐CDG impairs one of the first steps in the N‐glycosylation pathway and results in hypoglycosylation of glycoproteins. Congenital anomalies in PMM2‐CDG usually include facial anomalies, cerebellar hypoplasia, inverted nipples, abnormal fat pads, and arachnodactyly.^5^, ^6^ In 20% of cases, patients with PMM2‐CDG present with cardiac involvement, usually cardiomyopathy, arrhythmia, or pericardial effusion.^5^, ^7^, ^8^, ^9^ However, the association between PMM2‐CDG and congenital cardiac malformations is unclear.

We present a case of a patient with CDG and vascular ring anomaly and reviewed the literature on patients with CDG and concomitant CHD.

## 2 METHODS

### 2.1 Case presentation

Our patient is a 6‐year‐old girl who was referred for evaluation for psychomotor retardation, hypotonia, ataxia, inverted nipples, and a heart murmur. She was the third child born to healthy, nonconsanguineous Caucasian parents at 38 weeks' gestation via caesarean section with a birth weight of 4.2 kg (90‐95th percentile). Apart from the mother taking fluoxetine in early pregnancy and decreased fetal movement, the pregnancy was uneventful with no particular exposure to teratogenic substances.

During routine pediatric check‐up 1 week after birth, right‐sided torticollis, episodes of cyanosis and sleep apnea had been noted. At the age of 2 months, the patient presented with delayed development, failure to thrive, hypotonia, ataxia, muscle weakness, tongue fasciculation, and inverted nipples. Deep tendon reflexes were difficult to elicit. The palate was intact and normally arched, and no hearing problems were present at that time. Cranial ultrasound was normal and brain magnetic resonance imaging showed a myelination pattern at the lower limit of normal for her age. Electromyography showed no signs of motor neuron disease and myopathy. Spinal muscular atrophy gene sequencing as well as Prader‐Willi methylation and chromosome microarray were negative. Routine laboratory investigations revealed elevated total CK (240 U/L; standard range, <172 U/L) and elevated CK‐MB (10.4 ng/mL; standard range, 0.0‐6.0 ng/mL).

Due to worsening feeding problems, intermittent coughing and cyanosis, video fluoroscopy swallowing exam (VFSE) was performed at 3 months, which suggested vascular abnormality. Echocardiography revealed vascular ring anomaly with a right‐sided aortic arch, an aberrant left subclavian artery that originated from the descending aorta and a persistent foramen ovale (PFO). Cardiac segmental anatomy was otherwise normal. Ventricular hypertrophy or pericardial effusion was absent.

With the above clinical results, CDG‐I was suspected. Serum transferrin isoelectric focusing was performed at the age of 5 months and showed a type I pattern. At 8 months, subsequent whole exome gene sequencing revealed two PMM2‐CDG mutations in two different alleles: c.422G > A (p.R141H) and c.415G > A (p.E139K). No known pathogenic mutations related to CHD were found.

Family history revealed that the patient had one 2‐year‐older healthy brother and an older sister who passed away at 44 days after surgery for truncus arteriosus. The latter was suspected to have CDG based on a retrospective analysis of images and clinical features, but no whole exome gene sequencing was performed. Besides her older sister, there were no reported family members with either CHD or CDG. In addition, family history was negative for other genetic abnormalities and sudden cardiac death.

At 6 years of age, the patient suffers from global developmental delay, cerebellar atrophy, and peripheral neuropathy. She has no documented epilepsy. The patient did not undergo surgery and her vascular ring anomaly is followed up via VFSE and echocardiography. The PFO has closed naturally and other cardiac findings are normal. She was diagnosed with vision impairment (retinitis pigmentosa) with strabismus. Currently, she receives physical, occupational and speech therapy with favorable results.

### 2.2 Literature review

A PubMed database search was performed using the following terms: (CDG, type Ia OR PMM2 OR PMM2‐CDG OR PMM2 deficiency OR Jaeken syndrome OR carbohydrate‐deficient glycoprotein syndrome) AND (Heart OR CHD OR Cardiac malformation OR Artery OR Anomaly OR Vascular ring OR Tetralogy OR Coarctation OR Septal defect OR interrupted aortic arch OR stenosis OR Ebstein). All patients confirmed with PMM2 mutations or enzymatically confirmed PMM2 deficiency and with reported concomitant CHD, were included. Also, one previously unreported patient from our own database was included. This resulted in 14 PMM2‐CDG patients with CHD.

Patient demographics, diagnoses, and symptoms are presented in Tables 1 and 2. From 960 patients described in the literature, 14 had reported CHD.^10^, ^11^, ^12^, ^13^, ^14^, ^15^, ^16^, ^17^, ^18^, ^19^ The most common form of CHD in the patients of our literature review was tetralogy of Fallot (TOF) (5/14 patients, 35.7%), followed by persistent ductus arteriosus (PDA) (3/14, 21.4%) and truncus arteriosus (3/14, 21.4%). In contrast, these defects only represent 4.4%, 10.2%, and 1.0% of all CHD in the general population.^20^ The total prevalence of CHD of 1.5% (15/960) in PMM2‐CDG is only slightly higher than the 0.9% reported in the general population. This might not be significant, however, and might in part be explained by higher rates of consanguinity in families of patients with PMM2‐CDG. On the other hand, note has to be taken that minor manifestations of CHD might not have been reported in the literature of PMM2‐CDG patients, potentially leading to both an underestimation of total CHD prevalence and an overestimation of the relative abundance of major CHD in our literature study. Interestingly, the three patients with truncus arteriosus all had the p.E139K mutation, the same mutation observed in our patient and her sister with truncus arteriosus. The first of these patients had a mutation (c.470 T > C) on the other allele that also results in a dysfunctional protein with almost 0% enzymatic function, similar to the mutation reported in patient and her sister.^21^ The second patient was homozygous for the p.E139K mutation, due to uniparental maternal isodisomy for chromosome 16, where the *PMM2* gene is located. Uniparental isodisomy for chromosome 16 has previously been linked to CHD such as atrial septal defect, ventricular septal defect (VSD), and PDA; it is possible, however, that this association might be due to other mutations on this chromosome, rather than the *PMM2* mutations. CHD has also been reported in other genetic mutations underlying CDG, such as shown in the systematic review by Marques‐da‐Silva et al.^8^ The third patient had the mutation c.357C > A which results in only 25% enzymatic activity in comparison to the wild type.^22^

**TABLE 1:** Review of the literature about CHD in patients with PMM2‐CDG: patient details

Paper	Patient	Gender	Age at last follow‐up	Mutation 1^a^		Mutation 2^a^		Age at first signs of CDG	Age at diagnosis of CDG	Cardiac presentation	Surgical repair	CDG or CHD first	Deceased before last follow‐up	Cause of death	Failure to thrive	Growth retardation	Inverted nipples	Lipodystrophy	Other dysmorphic features
Al Teneiji et al^10^	Patient 6	Female	3.5 y	p.F144L	c.430 T > C	p.T237M	c.710C > T	at birth	3 mo	TOF	+	CHD	−	NR	+	NR	+	NR	NR
Damen et al^11^	Patient 3	Nr	NR	NR	NR	NR	NR	NR	NR	TOF	NR	NR	NR	NR	NR	NR	NR	NR	NR
Feldman et al^12^	Patient 1	Female	4 mo	NR	NR	NR	NR	at birth	8 mo	PDA, secundum ASD	NR	CHD	−	NR	NR	NR	+	+	+
Romano et al^13^	Patient 1	Female	15 y	p.E139K	c.415G > A	p.F157S	c.470 T > C	at birth	at birth	Truncus arteriosus	+	CHD	−	NR	+	+	+	+	NR
	Patient 2	Male	NR	p.E139K	c.415G > A^b^	p.F157S	c.470 T > C^b^	NR	PM	TOF	+	CHD	+	cardiac surgery	NR	NR	+	+	NR
	Patient 3	Female	14 y	p.E139K	c.415G > A	p.R141H	c.422G > A	at birth	at birth	TOF with absent pulmonary valve	+	CHD	−	NR	+	+	+	NR	NR
Rudaks et al^14^	Patient 2	Female	2 mo	p.R141H	c.422G > A	p.V231M	c.691G > A	at birth	2 mo	PDA	NR	CHD	+	heart failure	NR	NR	+	+	+
Serrano et al^15^	Patient 12	Female	6 y	p.P113L	c.338C > T	p.P113L	c.338C > T	3 mo	NR	PDA	−	NR	−	NR	NR	NR	NR	+	NR
	Patient 13	Male	11 y	p.E139K	c.415G > A	p.E139K	c.415G > A	at birth	NR	Truncus arteriosus	+	NR	−	NR	+	NR	NR	+	+
van de Kamp et al^16^	Patient 1	Male	7 d	p.F119L	c.357C > A	p.N59X	c.160_161insG	at birth	PM	PDA, PFO	−	CHD	+	cardiac function	NR	NR	+	NR	NR
Vermeer et al^17^	Case 1	Female	31 y	p.K51R	c.152A > G	p.K51R	c.152A > G	5 year	26 year	TOF	+	CHD	−	NR	NR	+	NR	NR	+
Verstegen et al^18^	Patient 2	Female	29 y	p.R21G	c.61A > G	p.R21G	c.61A > G	at birth	3 mo	Pulmonary artery stenosis	+	CHD	−	NR	NR	NR	NR	+	+
Wu et al^19^	Patient 1	Male	3 y	p.I153X	c.458_462 del	p.I132T	c.395 T > C	at birth	>12 mo	Secundum ASD	−	CHD	−	NR	+	+	+	NR	NR
Previously unreported	Patient 1	Male	7 y	p.E139K	c.415G > A	p.F119L	c.357C > A	NR	NR	Truncus arteriosus	+	CHD	−	NR	+	+	NR	NR	NR

*Note*: A minus sign (−) denotes the patient did not suffer from the pathology, a plus sign (+) denotes that it was present.

Abbreviations: ASD, atrial septal defect; CDG, CDG, congenital disorders of glycosylation; CHD, congenital heart defect; NR, not reported; PDA, patent ductus arteriosus; PFO, persistent foramen ovale; PM, postmortem; PMM2, phosphomannomutase 2; TOF, tetralogy of Fallot.

^a^ The mutations are first reported in the protein sequence and afterwards in the chromosome sequence.

^b^ Retrospective analysis of the patient confirmed symptoms of PMM2‐CDG and therefore this mutation is very likely.

**TABLE 2:** Other features of patients in the literature with CHD in patients with PMM2‐CDG

Paper	Patient	Hypotonia	Cerebellar atrophy	Psychomotor retardation	Developmental delay	Visual impairment	Nystagmus	Strabismus	Feeding difficulties	Hepatomegaly	Elevated liver enzymes	Protein‐losing enteropathy	Oedema	Recurrent infections	Hypothyroidism	Coagulopathy
Al Teneiji et al^10^	Patient 6	+	+	NR	+	+	NR	+	+	+	NR	NR	NR	NR	NR	+
Damen et al^11^	Patient 3	NR	NR	NR	NR	NR	NR	NR	NR	NR	NR	NR	NR	NR	NR	NR
Feldman et al^12^	Patient 1	NR	NR	NR	NR	NR	NR	NR	NR	NR	NR	NR	NR	NR	NR	NR
Romano et al^13^	Patient 1	+	+	+	+	+	+	+	+	NR	NR	NR	NR	NR	NR	NR
	Patient 2	NR	NR	NR	+	NR	NR	NR	NR	NR	NR	NR	NR	NR	NR	NR
	Patient 3	+	+	+	+	NR	+	NR	+	NR	NR	NR	NR	NR	NR	+
Rudaks et al^14^	Patient 2	NR	NR	NR	NR	NR	NR	NR	NR	+	NR	NR	+	NR	NR	+
Serrano et al^15^	Patient 12	NR	+	NR	NR	NR	NR	+	NR	NR	+	NR	NR	NR	NR	+
	Patient 13	NR	+	NR	NR	NR	NR	NR	NR	NR	NR	NR	NR	NR	NR	+
van de Kamp et al^16^	Patient 1	NR	NR	NR	NR	NR	NR	NR	NR	+	NR	NR	NR	NR	NR	+
Vermeer et al^17^	Case 1	+	+	+	NR	NR	+	NR	NR	NR	NR	NR	NR	NR	NR	NR
Verstegen et al^18^	Patient 2	NR	NR	NR	NR	NR	NR	NR	NR	NR	+	+	+	+	+	NR
Wu et al^19^	Patient 1	+	+	+	NR	NR	+	NR	NR	+	+	NR	NR	+	NR	+
Previously unreported	Patient 1	+	NR	+	+	+	+	−	+	−	−	−	−	−	−	−

*Note*: NR: not reported. A minus sign (−) denotes the patient did not suffer from the symptom, a plus sign (+) denotes that it was present.

Abbreviations: CDG, congenital disorders of glycosylation; CHD, congenital heart defect; PMM2, phosphomannomutase 2.

## 3 DISCUSSION

It is well known that glycosylation is essential in the organogenesis of all organs. As an example, neuropilin 1 (Nrp1) and neuropilin 2 (Nrp2) are glycosylated receptors that were originally discovered to regulate axonal migration and synaptogenesis in the central nervous system.^23^ Together with their ligand semaphorin 3A and coreceptors including plexin A2 and plexin D1, they are referred to the semaphorin/neuropilin/plexin (Sema/Nrp/Plxn) system. Recently, research has demonstrated an important role for this Sema/Nrp/Plxn system in vascular patterning and cardiac morphogenesis, especially the development of the cardiac septum and outflow tract. Mice deficient in Nrp1 have been shown to develop malformations of the great vessels and aortic arch.^24^ Plexin A2 and plexin D1 have been linked to TOF and truncus arteriosus, respectively.^25^, ^26^ In addition, inactivation of the transcription factor GATA‐6 in neural crest has been found to attenuate expression of semaphorin 3C, resulting in malformations of the cardiac outflow tract and aortic arch.^27^ It is plausible that impaired glycosylation resulting from the mutations seen in patients with CDG, might interfere with cardiac development by directly affecting posttranslational modification and thereby receptor function of Nrp1 and Nrp2. Studies have shown that inhibition of N‐glycosylation can indeed lead to impaired signaling function and increased internalization of the receptors.^28^, ^29^

Mutations underlying CDG might lead to disruptions in normal cardiac morphogenesis in yet another way. Incorrect glycosylation of proteins can lead to accumulation of unfolded or misfolded proteins in the endoplasmic reticulum (ER) and trigger the unfolded protein response (UPR), resulting in ER stress.^30^ ER stress has been linked to VSD, truncus arteriosus, and PDA in mice, and studies suggest that it is the main intracellular mechanism underlying maternal type 2 diabetes mellitus‐induced CHD.^31^ The UPR results in translational attenuation with decreased expression of BMP4, NKX2.5, and GATA5, all of which have been associated with CHD.^32^ In conclusion, defects of glycosylation in CDG might increase the rate of CHD in two proposed ways: either by directly affecting essential proteins in cardiac morphogenesis and vascular patterning, or indirectly by inducing ER stress and its detrimental effects on developing tissues. More basic research is required to further elucidate the potential molecular relationship between CDG and CHD, as well as the role of glycosylation in various congenital malformations.^33^

## 4 CONCLUSION

We present a PMM2‐CDG patient with a vascular ring anomaly: a right‐sided aortic arch and an aberrant left subclavian artery. In our review, we found that 1.5% of patients with PMM2‐CDG reported in the literature had concomitant CHD which is slightly higher than the general population risk of 0.9%. In most cases with delayed diagnosis of CDG. We suggest that CDG should be in the differential diagnosis of patients presenting with CHD in addition to dysmorphic features, psychomotor retardation, growth, and developmental delay.

## CONFLICT OF INTEREST

The authors declare no conflict of interest.

## AUTHOR CONTRIBUTIONS

**Zhen Qian**: conception, data collection, drafting the article, and revising the article for important intellectual content. **Jef Van den Eynde**: data collection, drafting the article, and revising the article for important intellectual content. **Zhen Qian** and **Jef Van den Eynde** contributed equally to the manuscript. **Stephane Heymans**: revising the article for important intellectual content. **Luc Mertens**: revising the article for important intellectual content. **Eva Morava**: providing information of the case, conception, drafting the article, and revising the article for important intellectual content. All the authors have seen and approved this paper. This article is the original work of all the authors listed and has not been published elsewhere and is not under consideration elsewhere.

## STATEMENT OF CONSENT

The authors confirm that written consent for submission and publication of this case report including images and associated text has been obtained from the patient in line with COPE guidance.
